# Heterozygous females from a rat model for creatine transporter deficiency reveal altered behavioral response to stressors, normal body weight and slight metabolic changes

**DOI:** 10.3389/fnins.2025.1520550

**Published:** 2025-04-29

**Authors:** Lara Duran-Trio, Marc Lanzillo, Dunja Simicic, Clothilde Roux-Petronelli, Stephen J. Bruce, Carmen Sandi, Cristina Cudalbu, Olivier Braissant

**Affiliations:** ^1^Service of Clinical Chemistry, University of Lausanne and Lausanne University Hospital of Lausanne, Lausanne, Switzerland; ^2^Centre d'Imagerie Biomedicale (CIBM), Ecole Polytechnique Fédérale de Lausanne (EPFL), Lausanne, Switzerland; ^3^Brain Mind Institute, Ecole Polytechnique Fédérale de Lausanne (EPFL), Lausanne, Switzerland; ^4^Animal Imaging and Technology, Ecole Polytechnique Fédérale de Lausanne (EPFL), Lausanne, Switzerland

**Keywords:** creatine, creatine transporter deficiency, SLC6A8, female, X-linked disorder, behavior, inherited metabolic disease

## Abstract

Creatine (Cr) is an organic acid essential for recycling ATP, important in tissues with high energy demand such as muscle or brain. Cr is synthesized in a 2-step pathway by the enzymes AGAT and GAMT, and transported by SLC6A8 (also called CrT). Cerebral Cr deficiency syndromes (CCDS), due to AGAT, GAMT or CrT deficiencies, are metabolic diseases characterized by brain Cr deficiency, causing a range of clinical features such as severe neurodevelopmental delays and intellectual disability, behavioral disturbances, motor dysfunction and epilepsy. Among CCDS, the X-linked CrT deficiency (CTD) is the most prevalent with no efficient treatment so far. Increasing number of human and animal studies contributes to the understanding of CTD pathology, its diagnosis and treatment, and the roles of Cr and CrT. However, most of them are focused in males and little is known about female carriers and how CrT deficiency affect them. In order to increase knowledge in female sex and roughly explore the relationship with SLC6A8 gene dosage, we present the first characterization of females' *Slc6a8*^*Y*389*C*^ rat model of CTD using both heterozygous and homozygous females. Brain Cr deficiency was found in all homozygous females, while heterozygous ones showed broad variability in brain Cr levels. Elevated and slightly elevated urinary Cr/Crn ratio were present in homozygous and heterozygous females, respectively. Reduced body weight, muscular mass and locomotor activity were hallmarks of homozygous, but not heterozygous, females. However, in contrast to *Slc6a8*^*Y*389*C*^ KI males, spontaneous alternation and grooming behaviors were not affected in any type of *Slc6a8*^*Y*389*C*^ mutant female rats. Interestingly, both *Slc6a8*^*Y*389*C*^ mutant female rats exhibited behavioral abnormalities such as increased prevalence of altered behavioral response to handling, being more frequent in homozygous female rats. Moreover, heterozygous females presented increased anxiety-like behavior to novelty in Open Field Novel Object test and altered behavioral response with increased locomotor activity in response to light as stressor in the Light Dark Box test. These results are coherent with the limited data from CTD human female carriers, validating the *Slc6a8*^*Y*389*C*^ rat females as a promising tool to better understand CTD in female sex. They also provide new insights about CTD pathology, revealing sex and zygotic phenotypic differences, highlighting the importance of including females in the study of CTD.

## 1 Introduction

Creatine (Cr) is a nitrogenous organic acid essential for recycling ATP, important in tissues with high energy demand such as muscle or brain (Hanna-El-Daher and Braissant, 2016; Wyss and Kaddurah-Daouk, 2000). Cr is either taken up from the diet or endogenously synthesized in a 2-step pathway by the enzymes AGAT (arginine-glycine amidinotransferase) and GAMT (guanidinoacetate methyltransferase). It is specifically transported into the cells by SLC6A8 (also called CRT, CreaT or CT1, henceforth CrT), and spontaneously broken down in creatinine (Crn) (Wyss and Kaddurah-Daouk, 2000).

Cerebral Cr deficiency syndromes (CCDS), due to AGAT, GAMT or CrT deficiencies, are metabolic diseases characterized by brain Cr deficiency, causing a range of clinical features such as severe neurodevelopmental delays, intellectual disability (ID) with speech delay, behavioral disturbances, motor dysfunction, and seizures (Item et al., 2001; van de Kamp et al., 2013; Salomons et al., 2001; Schulze and Braissant, 2017; Stöckler et al., 1994; Stöckler-Ipsiroglu and van Karnebeek, 2014). Among CCDS, the X-linked CrT deficiency (CTD) is the most prevalent one, being a common cause of X-linked ID in males (Rosenberg et al., 2004), with no efficient treatment so far.

In addition to clinical studies, preclinical models of CTD have been generated and recapitulate features similar to those of patients, contributing to the understanding of CTD pathology and the development of therapies (Baroncelli et al., 2014; Duran-Trio et al., 2021; Fernandes-Pires and Braissant, 2022; Ghirardini et al., 2021; Skelton et al., 2011; Stockebrand et al., 2018). However, most of the human and animal studies are focused in males and little is known about female carriers and how CrT deficiency affect them.

Preclinical data showed *Slc6a8*^−/+^ knock-out (KO) heterozygous female mice with partial cognitive deficiency and partial brain Cr deficiency (Hautman et al., 2014). On the other hand, the limited clinical data about CTD in females describes heterozygous carriers whose clinical symptoms range from asymptomatic/mild to severe phenotype resembling males' one, metabolic biomarkers (e.g., brain Cr levels assessed by proton magnetic resonance spectroscopy, ^1^H-MRS, or urinary Cr/Crn ratio) ranging from normal to abnormal parameters, and relatively high treatment efficacy by Cr supplementation (Bruun et al., 2018; van de Kamp et al., 2013; Nielsen et al., 2023).

This broad picture has been justified by the residual activity of CrT and the variability in the mosaic expression pattern of functional/dysfunctional CrT due to lyonization (also called random X-chromosome inactivation or X-inactivation).

Noteworthily, despite sex/gender differences in increasing number of pathologies (Mauvais-Jarvis et al., 2020), many clinical CTD female cases have been found through family testing, reflecting clear diagnostic bias in female sex, and thus, preventing them from accessing the potential benefits of Cr supplementation. Turning the tables, it is possible that such scarcity of clinical and preclinical studies in females is indeed influencing the understanding of CTD pathology, leading to underestimation or misdiagnosis of CTD in female sex.

Therefore, in order to increase the understanding of the pathology, and provide valuable data for CTD diagnosis in female sex, we present here the first characterization of females from the *in vivo* rat model of CTD, the *Slc6a8*^*Y*389*C*^knock-in rat holding an identic patient's missense mutation abolishing CrT activity (Duran-Trio et al., 2021, 2022; van de Kamp et al., 2013). In particular, we aimed to roughly explore whether there is a relationship between SLC6A8 gene dosage and CTD phenotype in females, by using wild-type, *Slc6a8*^*x*/*xY*389*C*^ heterozygous and *Slc6a8*^*xY*389*C*/*xY*389*C*^ homozygous female rats (fWT, fHE, and fHO henceforth, respectively). Thus, fHE rats are expected to show phenotypic traits in between those from fWT and fHO rats, with quite variability among rats due to lyonization (considering all the possibilities of stochastic inactivation: skewed toward one allele, toward the other, and not skewed).

We carried out a similar study than those for male *Slc6a8*^*Y*389*C*^rats' characterization: quantification of metabolites related with Cr in different tissues and body liquids, analysis of muscle phenotype, and conduction of used and new sets of behavioral tests, related with behavioral features and phenotypic traits described in CTD patients.

## 2 Methods

### 2.1 Animals

Wild-type (WT), *Slc6a8*^*Y*389*C*^ knock-in [KI, (Duran-Trio et al., 2021)] heterozygous (*Slc6a8*^*x*/*xY*389*C*^) and homozygous (*Slc6a8*^*xY*389*C*/*xY*389*C*^) female rats (fWT, fHE, and fHO henceforce, respectively) were obtained from crossbreedings between fHE and WT or *Slc6a8*^*Y*389*C*^ KI males. Littermates of 3–4 months of age were used for all the experiments. Animals were genotyped and housed as previously described (Duran-Trio et al., 2021). All experiments were performed with approval of Canton de Vaud veterinary authorities (VD-3284) in accordance with the Swiss Academy of Medical Science and followed the ARRIVE Guidelines 2.0. Efforts were made to minimize stress and number of animals used.

### 2.2 *In vivo* ^1^H-MRS in CNS

*In vivo* proton magnetic resonance spectroscopy (^1^H-MRS) at high magnetic field (9.4T) was performed in CNS as previously described (Duran-Trio et al., 2021). 4 fWT, 8 fHE and 4 fHO *Slc6a8*^*Y*389*C*^ female rats were used.

### 2.3 Tissue and liquids collection, measures of Cr, GAA, and Crn, and histology

Tissue and liquids (plasma, urine) were collected as described (Duran-Trio et al., 2021). Measures of Cr and GAA were performed by liquid chromatography coupled to double mass spectrometry (LC/MS-MS) as described (Braissant et al., 2010; Duran-Trio et al., 2021). Measure of Crn was performed on a COBAS 8000 automate (Roche, Switzerland). Histology of muscle tissue was done as described (Duran-Trio et al., 2022).

### 2.4 Behavioral tests

Female rats were subjected to a battery of tests described in Duran-Trio et al. (2021; 2022): open field novel object (OFNO), elevated plus maze (EPM), light-dark box (LDB) and Y-maze spontaneous alternation. All tests were recorded with a videocamera and analyzed offline. A vaginal smear was done 2–3 h after each test (see below), and rats remained undisturbed 1–3 days between tests. To minimize circadian effects, animals were tested at the same time interval of the day.

### 2.5 Open field novel object, locomotion, rearing up and grooming behavior in an open field

For OFNO test, rats were introduced in a circular open arena (1 m diameter, black PVC) for 10 min (OF phase). Then, a novel object was located in the middle of the open arena and the rat was left undisturbed for other 5 min (NO phase). Spontaneous behavior was recorded with a videocamera. Average velocity, total distance moved, and time spent moving or not moving (considering thresholds of 2 and 1.75 cm/s, respectively) were calculated using EthoVision tracking software per each phase. Additionally, arena was virtually segmented in three concentric areas (“center” is the 12.5 cm radius region in the center of the arena, “wall” is the external region of 12.5 cm radius from the wall, and “intermediate” is the resting region between them) and cumulative duration spent in each one was analyzed with EthoVision. Index were calculated as the relative difference between NO and OF phases [Index = (NO – OF)/OF]. “Index Dur” is from cumulative duration (in %) spent in each region and phase, and “Index Mov” is from cumulative duration moving (in %) in each phase.

OF phase (10 min) recordings were used for the analysis of grooming behavior, rearing supported (standing on hind limbs and one or two superior extremities) and unsupported (no superior limb used). Such behaviors were hand scored with The Observer XT software.

### 2.6 Elevated plus maze

The elevated plus maze (EPM) test consists of two opposing open arms (45 × 10 cm, 16 lux) and two opposing enclosed arms (45 × 10 × 50 cm, 4 lux) that extend from a central platform (10 × 10 cm, 11 lux), elevated 65 cm above the floor. Rats were placed individually on the central platform facing an enclosed arm and were allowed to freely explore the maze for 5 min. The behavior of each rat was monitored using a videocamera, and the movements of the rats were automatically registered and analyzed with EthoVision^®^ tracking software. Entry into an arm was defined as entry of all four paws into one arm. Cumulative duration and number of times the animal spent in each type of arm were analyzed, as well as head dipping behavior (looking over the edge of an open arm or the central platform) and the latency to the first open arm.

### 2.7 Light-dark box

The light-dark box apparatus consists of two compartments (40 × 40 × 50 cm each), one with black walls and floor, illuminated at 20 lux, and the other with white walls and floor at 160 lux. The two compartments are connected by a small opening (8 × 10 cm high) located in the center of the partition at floor level. During the test the animals were individually placed in the middle of the white compartment facing away from the opening of the dark box and were recorded with a videocamera for 5 min. The time spent in the black compartment, the number of entries into the white compartment and the latency to the black compartment were scored with EthoVision^®^ tracking software. Some videos were hand-scored blindly with The Observer™ software to check the automatic analysis.

### 2.8 Y-maze spontaneous alternation

Same test and procedure as previously described (Duran-Trio et al., 2021).

### 2.9 Vaginal smearing and determination of estrous cycle

A tip of 1 ml with 200 μl of saline (0.9 % NaCl) was introduced in the vagina (no more than 0.5 cm deepness) of an immobilized rat (tail up to expose the genitals). The liquid was pipetted in and out 2–3 times, then extended on a slide and let dried in a heat pad. Hematoxylin-eosin staining was performed as described (Duran-Trio et al., 2022). Using an OLYMPUS microscope, estrous phases were identified according to Cora et al. (2015). For the analysis, 3 factors were considered (“diestrous”, “estrous”, and “rest” where proestrous and metestrous were grouped together).

### 2.10 Imaging and quantifications

Images of stained muscles were taken on an OLYMPUS microscope; cross-sectional areas and minimum Feret diameter were obtained with ImageJ (Duran-Trio et al., 2022).

### 2.11 Statistical analysis

All statistical analysis and graphs were conducted with R-3.5.1 (R Core Team, 2018). A Shapiro test was used to assess the normality of each sample. Mann-Whitney test with Bonferroni correction was used for pair comparisons among genotypes when normality was rejected. For normal distributions, we used one-way analysis of variance (ANOVA) followed by Tukey *post hoc* test for pair comparisons among genotypes, or *t-*test to address the significance between a genotype with respect to the value expected by chance.

For behavioral tests, generalized linear mixed models blocking estrous cycle [diestrus, estrous, rest=(proestrous+metestrus)] as random factor were used to analyze the differences among genotypes [package nlme (Pinheiro et al., 2015)], and obtained coefficients (mean and standard error of the mean) were used for graphs. Graphs were done using ggplot2 package (Wickham, 2016). *P*-values are reported in figure legends, and statistical significance was considered at *P* < 0.05.

## 3 Results

### 3.1 *Slc6a8^*Y*389*C*^* female rats are viable and fertile

Both *Slc6a8*^*Y*389*C*^ fHE and fHO female rats are viable and fertile, although the later ones showed reduced fertility (giving only half of the offspring) when crossed with *Slc6a8*^*Y*389*C*^ KI males.

### 3.2 *Slc6a8^*Y*389*C*^* homozygous, but not heterozygous females, present reduced body weight gain

**Figure 1:**
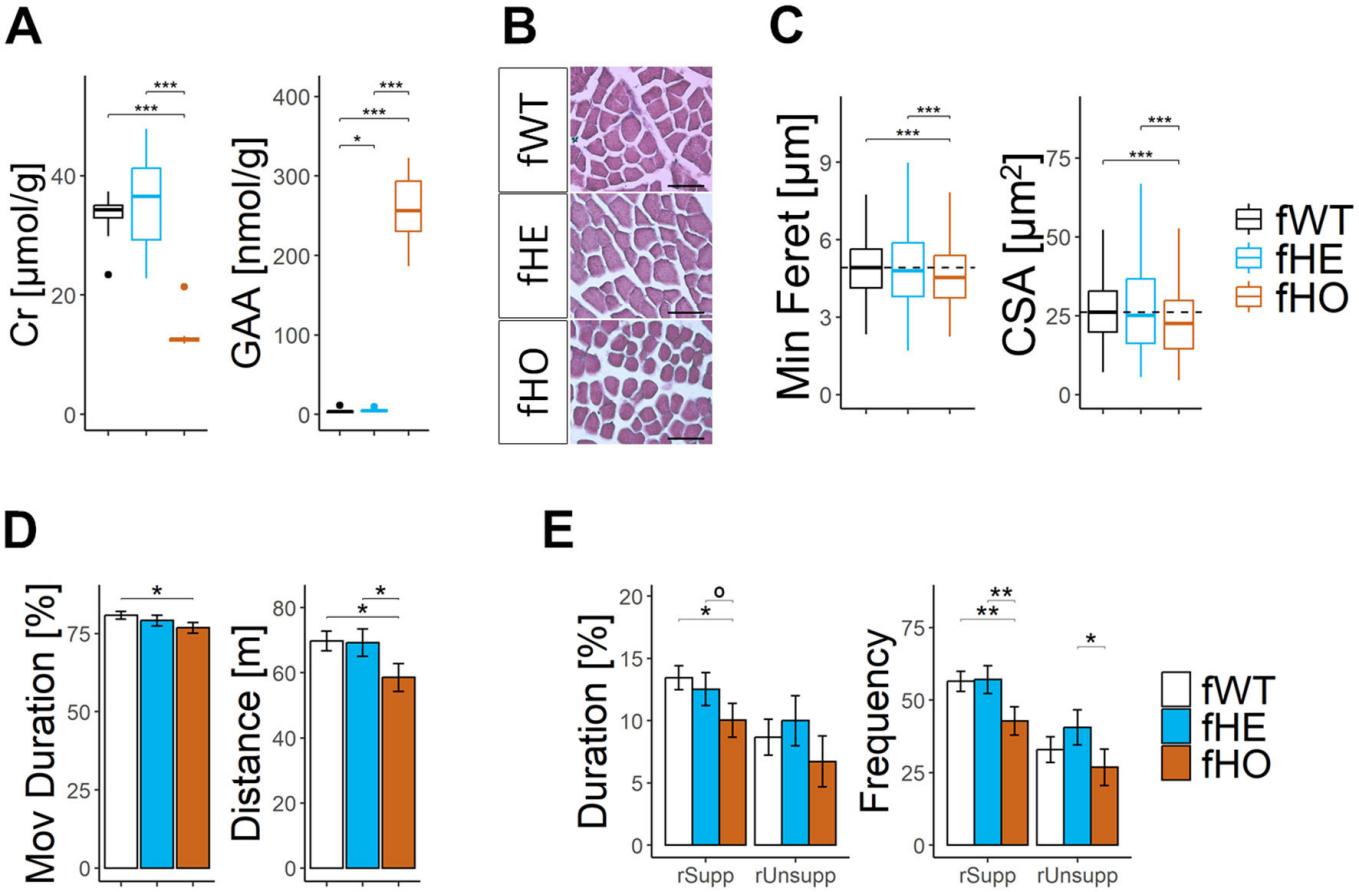
Homozygous females show strong CrT deficiency with severe brain creatine deficiency and reduced body weight, while heterozygous females show CrT deficiency with broad effects on brain creatine deficiency and normal body weight. **(A)** Line-plot showing body weight (in grams) along age (in weeks) per each genotype. Error bars represent standard deviation. No significant differences were found between fWT and fHE rats, but significant differences from 6wo were found between fHO and fWT (as “*”) or between fHO and fHE rats (as ”ª”). 1way-ANOVA and Tukey *post hoc*; 16 fWT, 21 fHE and 13 fHO. **(B)** Representative picture of each genotype at 13 weeks-old. Rectangle is 12.5 × 28 cm. **(C)** Boxplot of urinary Cr/Crn. 15 fWT, 16 fHE and 15 fHO. Pair comparisons were made using Mann-Whitney test with Bonferroni correction. **(D)** Representative 9.4T ^1^H-MRS spectra in the cerebellum of 1 fWT, 2 fHE and 1 fHO. Note the strong decrease of the Cr and PCr peaks in the fHO and the differences in the amplitude of such peaks in fHE. The localization of the measured voxel is presented on top of the panel. **(E)** Boxplots of ^1^H-MRS Cr and/or PCr concentrations in cerebellum (Cb) and hippocampus (Hp) in each genotype. *P-values* from 1way-ANOVA and pair comparisons using Tukey *post hoc* test are shown in the table above. 3 fWT, 8 fHE, and 3 fHO. °*P-value* < 0.1, *P-value < 0.05, **P-value < 0.01, ***P-value < 0.001.

Reduced body weight gain is characteristic of CTD male patients and rodent models of CTD, including the KI rat males (Baroncelli et al., 2014; Duran-Trio et al., 2021; van de Kamp et al., 2013; Miller et al., 2019; Skelton et al., 2011; Stockebrand et al., 2018). In comparison with fWT, *Slc6a8*^*Y*389*C*^ fHE did not show significant differences in body weight gain along time, while *Slc6a8*^*Y*389*C*^fHO presented significant less body weight gain along time from 6 weeks-old on (peri-adolescence) (Figures 1A, B).

### 3.3 Urinary Cr/Crn ratio is elevated in *Slc6a8^*Y*389*C*^* heterozygous and homozygous female rats

Due to CrT deficiency in kidney, *Slc6a8*^*Y*389*C*^ KI male rats present elevated Cr/Crn (as CTD male patients) and GAA/Crn ratios in urine in comparison with WT male rats (Duran-Trio et al., 2021). Given that fHO rats also lack functional CrT, we expected that Cr will be lost in the urine too, thus showing elevated urinary Cr/Crn ratio. On the other side, cells from fHE rats will or will not express a functional CrT, depending on which X-chromosome is silenced. Therefore, taking into account the mosaics' variety, a broad spectrum from normal to elevated Cr levels in urine would be theoretically expected.

Indeed, fHO rats presented a significant increase in both urinary Cr and GAA concentrations and a significant decrease of Crn levels (+600%, +300% and −67% vs. fWT, respectively; Supplementary Figure 1A), resulting in significant elevated urinary Cr/Crn and GAA/Crn ratios in comparison with fWT (× 148 and × 16, respectively; Figure 1C and Supplementary Figure 1A).

On the other hand, compared to fWT, fHE rats presented significant elevated urinary Cr levels (x7) and slight but not significant increase in GAA and Crn concentration (Supplementary Figure 1A), resulting in a significant increase in urinary Cr/Crn (x11) ratio but no significant changes in GAA/Crn ratio (Figure 1C and Supplementary Figure 1A).

### 3.4 Plasma Cr levels decrease in both *Slc6a8^*Y*389*C*^* mutant females while GAA levels increase

In comparison to fWT rats, both fHE and fHO rats exhibited significant changes in plasmatic Cr and GAA levels, decreasing the first one and increasing the later one (Supplementary Figure 1A). However, plasmatic Crn levels only decreased in fHO (Supplementary Figure 1A).

### 3.5 *Slc6a8^*Y*389*C*^* homozygous females show strong brain Cr deficiency while heterozygous females show variability in brain Cr concentration

CTD male patients are characterized by brain Cr deficiency and are diagnosed using ^1^H-MRS. This is also the case in CTD rodent models, including the *Slc6a8*^*Y*389*C*^ rat (Duran-Trio et al., 2021). High magnetic field 9.4 T ^1^H-MRS spectra from cerebellum (Figure 1D) and hippocampus (data not shown) systematically showed much lower peaks corresponding to Cr and PCr in *Slc6a8*^*Y*389*C*^ fHO compared to those of fWT ones, while a broad variety of peaks heights was observed in fHE spectra. Levels of Cr and PCr together (Cr+PCr) were significantly reduced in fHE rats compared to those of fWT; and those of fHO (−73% and −84% vs. fWT in cerebellum and hippocampus, respectively) to those of fWT as well as to those of fHE (Figure 1E).

### 3.6 *Slc6a8^*Y*389*C*^* homozygous females show metabolic changes in brain

Taking advantage of the high resolution 9.4T ^1^H-MRS measures to quantify potential metabolic changes in the brain of *Slc6a8*^*Y*389*C*^ rats, we observed that fHO, but not fHE, presented significant differences in some metabolites' concentrations in comparison with those of fWT ones (Supplementary Figure 1B). Gln, Glu, and Gln+Glu were increased in cerebellum, while NAA levels were increased in both cerebellum and hippocampus, and NAA+NAAG only augmented in hippocampus.

### 3.7 *Slc6a8^*Y*389*C*^* homozygous, but not heterozygous, female rats show reduced muscle Cr concentration and myocyte size

**Figure 2:**
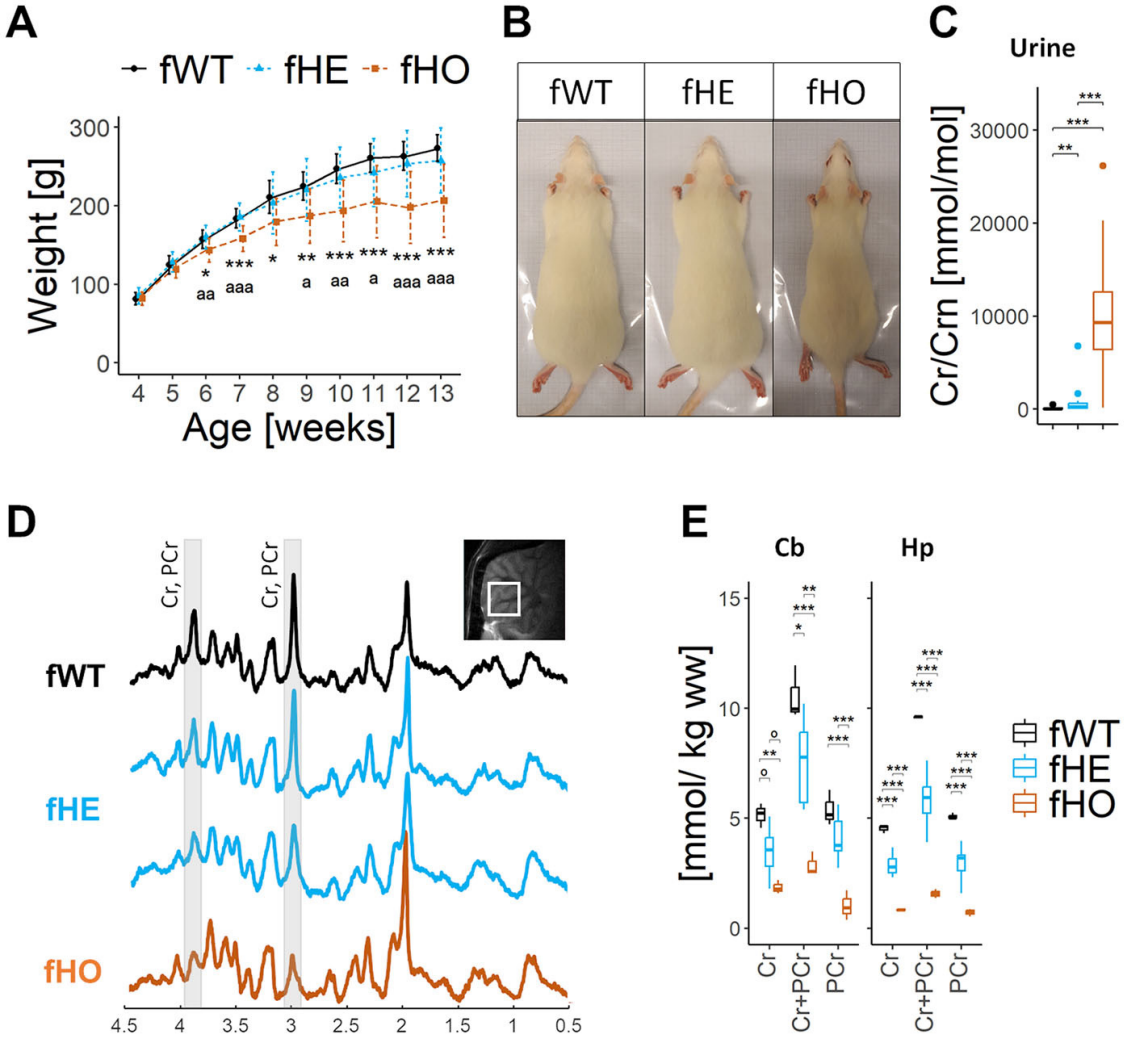
Homozygous, but not heterozygous, female rats show altered muscle phenotype and reduced locomotor activity. **(A)** Boxplots of muscle Cr and GAA concentrations. Pair comparisons using Mann-Whitney test with Bonferroni correction. 12 fWT, 12 fHE, and 11 fHO. **(B)** Representative microscopic pictures of hematoxylin/eosin staining in transversal sections of quadriceps muscle. Scale bar = 15 μm. **(C)** Boxplots of cross-sectional area (CSA) and minimum Feret diameter (Min Feret) from myocytes of quadriceps muscle. Pair comparisons using Mann-Whitney test with Bonferroni correction. 4 fWT, 11 fHE and 4 fHO (128–194 measurements per WT female, 124–281 per fHE, and 156–208 per fHO; 659, 2,000, and 721 total myocytes per genotype, respectively). **(D)** Barplots of total distance and relative time moving (Mov Duration) in the open field (OF) test. Pair comparisons with Nested ANOVA blocking estrous cycle as a random factor. 11 fWT, 12 fHE, and 11 fHO. **(E)** Barplots of cumulative duration and frequency of supported and unsupported rearing up (rSupp and rUnsupp, respectively), in the open field (OF) test. Pair comparisons with Nested ANOVA blocking estrous cycle as a random factor. 11 fWT, 12 fHE, and 11 fHO. °*P-value* < 0.1, *P-value < 0.05, **P-value < 0.01, ***P-value < 0.001.

Muscle Cr levels significantly decreased while GAA levels significantly increased in fHO rats compared to those of fWT or fHE ones (Figure 2A). However, only muscle GAA levels from fHE rats significantly differed from those of fWT ones.

Furthermore, myocytes from fHO rats exhibited significantly smaller minimum Feret diameter and cross-sectional area compared to those of fWT or fHE ones (Figures 2B, C). Myocytes from fHE rats presented similar size than those of fWT rats.

### 3.8 *Slc6a8^*Y*389*C*^* homozygous, but not heterozygous, female rats present reduced locomotor activity

Locomotor activity was evaluated during free exploration in an open field. When compared to fWT, fHO rats showed significant less distance moved, less cumulative duration moving, less cumulative duration doing supported rearing and less frequency of supported and unsupported rearing in comparison with fWT rats (Figures 2D, E). In contrast, fHE rats showed similar performance as fWT rats.

### 3.9 *Slc6a8^*Y*389*C*^* mutant females exhibit normal performance in a working memory/attention task and normal grooming behavior

Our previous findings showed that *Slc6a8*^*Y*389*C*^ KI rat males exhibited bad performance in a working memory and attention task (the Y-maze alternation test) and increased grooming behavior (Duran-Trio et al., 2021).

**Figure 3:**
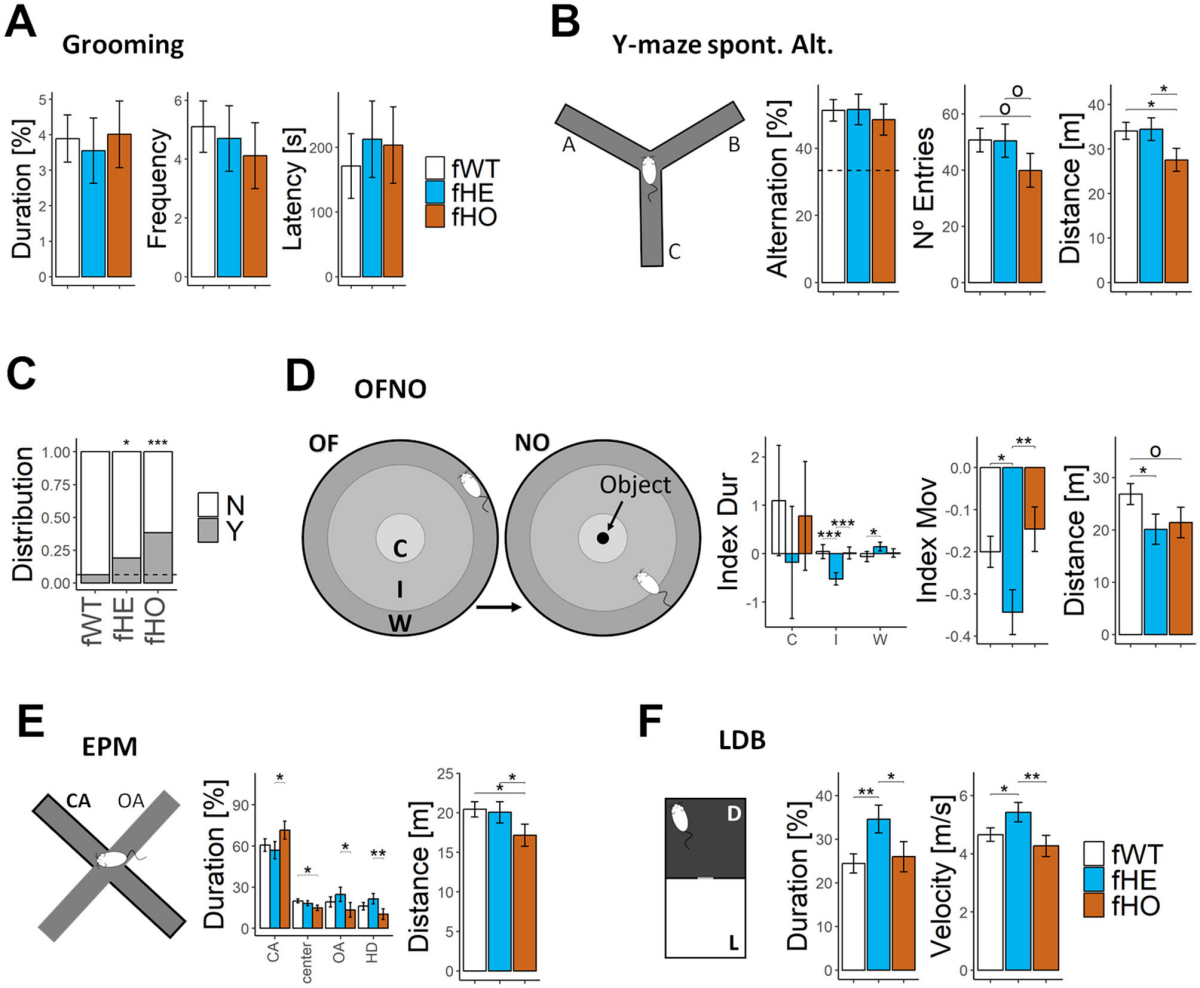
Mutant females show altered behavioral response to stressors, but similar performance in Y-maze spontaneous alternation test and grooming behavior than WT females. **(A)** Barplots of cumulative duration (in %), frequency and latency of grooming behavior in an open field. 11 fWT, 12 fHE, and 11 fHO. **(B)** Y-maze spontaneous alternation test's infographic **(left)** and barplots **(right)** showing percentage of alternation, number of entries and distance moved in Y-maze spontaneous alternation test. All genotypes presented significant difference with respect to the value expected by chance in percentage of alternation (100/3, horizontal dot line; *p* < 0.001, *p* < 0.001, and *p* = 0.002 for fWT, fHE, and fHO, respectively, *t-*test), but no among them. 12 fWT, 12 fHE, and 11 fHO. **(C)** Barplot of rats' relative distribution presenting (Y) or not (N) behavioral abnormalities in response to handling at the onset of adolescence (7–8 wo). Two-sided Exact binomial test evaluating differences with respect to the fWT group (*p* < 0.05 and *p* < 0.001 for fHE and fHO, respectively). 16 fWT, 21 fHE, and 13 fHO. **(D)** OFNO test's infographic **(left)** and barplots **(right)** showing the relative difference in cumulative duration between NO and OF phases spent per each region (C = center, I = intermediate, W = wall; Index Dur) or spent moving (Index Mov), and of the total distance moved in the NO phase (OF phase is shown in Figure 2D). 11 fWT, 10 fHE, and 10 fHO. **(E)** EPM test's infographic **(left)** and barplots **(right)** of cumulative duration spent in each region (CA = closed arm, center, OA = opened arm, HD = head dipping in the OA), and total distance moved. 12 fWT, 12 fHE, and 11 fHO. **(F)** LDB test's infographic **(left)** and barplots **(right)** of cumulative duration spent in the light compartment and average velocity. 12 fWT, 11 fHE, and 8 fHO. Error bars represent standard error of the mean. Pair comparisons among genotypes were performed with Nested ANOVA blocking estrous cycle as a random factor. °*P-value* < 0.1, *P-value < 0.05, **P-value < 0.01, ***P-value < 0.001.

However, neither fHE nor fHO rats showed significant differences with respect to fWT rats in the total duration, frequency or latency of grooming behavior evaluated in similar conditions (Figure 3A).

Additionally, in the Y-maze task, there were no significant differences among genotypes in the number of entries, although fHO rats showed a tendency to reduce it and significant less distance moved compared to both fWT and fHE rats. Noteworthily, fHE and fHO rats showed no significant differences in percentage of alternation in comparison with fWT rats (Figure 3B), suggesting for all genotypes a good performance in the task (since their values significantly differed from the value expected by chance: 100/3).

### 3.10 *Slc6a8^*Y*389*C*^* mutant female rats exhibit altered behavioral response to stressors

We observed that, at the onset of adolescence (7–8 weeks-old), both fHE and fHO rats significantly started displaying abnormal behavior (Figure 3C) when handling them in comparison with fWT rats (around 6% for fWT, while 19% and 38% for fHE and fHO, respectively). They screamed, trying to flee, increasing locomotion and jumping out. At early adulthood (11 weeks-old), both fHE and fHO needed longer habituation time (doubling the number of days in some cases) to handle them without signs of stress before performing the behavioral tests. Additionally, we also observed that both mutant female rats, specially fHO rats, were more prone to bite both other rats in the same cage and human researchers.

In order to evaluate rats' behavioral response to stressors, anxiety-like behavior and locomotion were assessed in different tests that involve 3 different types of stressors, including novelty (OFNO), openness (EPM) and light (LDB).

In the OFNO test, there were no significant differences among genotypes in the OF phase except for fHO rats who moved less distance in comparison with fWT and fHE rats (Figure 2D and Supplementary Table 1). Due to their natural aversion to open spaces, rats explored freely the open field spending longer time close to the wall (nearly 70% of the time, 3% on the arena center). However, after locating the novel object in the center of the arena (NO phase), fWT rats decreased their time spent in the wall zone (to 65%) by increasing their time exploring the novel object in the center (to 5%) and surroundings (Figure 3D and Supplementary Table 1). In contrast, fHE rats significantly raised their time spent in the wall zone (to 85%) and reduced it nearby the novel object. Additionally, fHE rats significantly decreased their time moving and the total distance moved in comparison with fWT females. In contrast, fHO females did not show significant differences with respect to fWT rats. These results indicate that fHE females present increased anxiety-like behavior to novelty.

In the EPM test, due to rodents' natural aversion to open spaces, fWT rats stayed longer time in the closed arms than in the opened arms (60 vs. 19% of the time). Interestingly, although both fHE and fHO rats did not show significant differences in the duration spent in each compartment in comparison with fWT ones (Figure 3E), they significantly exhibited an opposite pattern in the exploration of closed and open spaces: in comparison with fHE rats, fHO rats spent longer time in the closed arm and less (around the half of the time) in the open arm and doing head dipping. These results indicate that fHE and fHO rats display normal anxiety-like behavior in this test, being, respectively, at the anxiolytic and anxious extremes of a wild-type phenotype.

In LDB test, due to rodents' natural aversion to illuminated places, fWT rats spent only around 25% of the time in the light compartment and 75% in the dark one. A similar pattern was presented by fHO rats, but fHE rats stayed significantly longer time in the light compartment and moved with higher velocity than fWT and fHO rats (Figure 3F). These results indicate that fHE rats present altered behavioral response and hyperactivity to light as stressor.

In summary, both mutant *Slc6a8*^*Y*389*C*^ female rats present behavioral abnormalities in response to stressors, namely increased or decreased anxiety-like behavior with or without hyperactivity, depending on the trigger and the zygosity.

## 4 Discussion

Under the assumption of less pronounced CTD phenotypic and pathological traits despite the evidences of sex/gender differences in many pathologies (Mauvais-Jarvis et al., 2020), human and animal model studies in female sex are underrepresented. However, lack of studies in females may influence the underestimation or misdiagnosis of CTD in females.

In order to increase the understanding of the pathology and providing useful data for CTD diagnosis in female sex, we present here the first characterization of females from the *in vivo* rat model of CTD, the *Slc6a8*^*Y*389*C*^knock-in rat holding an identic patient's missense mutation abolishing CrT activity (Duran-Trio et al., 2021, 2022; van de Kamp et al., 2013). Carrying out similar study than those for *Slc6a8*^*Y*389*C*^male rat's characterization (related with behavioral features and phenotypic traits described in CTD patients), by using wild-type (fWT), *Slc6a8*^*xY*389*C*/*x*^ heterozygous (fHE) and *Slc6a8*^*xY*389*C*/*xY*389*C*^ homozygous (fHO) rats, we roughly explored the relationship between SLC6A8 gene dosage and CTD phenotype in female rats.

Brain Cr deficiency, the main characteristic of CTD pathology, was observed in both fHE and fHO rats. Strong Cr deficiency in fHO rats was expected since they cannot express any functional CrT in comparison with fHE rats. On the other hand, the broad range of Cr levels in fHE brains could be explained by the supposed variability in the mosaic expression patterns of functional/dysfunctional CrT caused by random lyonization. These results agree with the brain Cr deficiency found in heterozygous females from a CTD mouse model (Hautman et al., 2014) and in female CTD carriers (Nielsen et al., 2023). Noteworthily, fHE's range overlapped a little with fWT's in cerebellum. This result is in consonance with clinical studies showing female carriers with brain Cr levels in a normal range (Nielsen et al., 2023), and highlights that every brain region might display a different picture in CTD pathology, warning about the possibility of false negatives.

Indeed, the analyzed brain regions showed different Cr levels in WT conditions and responded differentially to CrT deficiency. Despite cerebellum showed the highest Cr levels in WT conditions, hippocampus presented the greatest/largest absolute and relative Cr depletion (and with less variability) in conditions of CTD. Similar picture appeared in KI male rats (Duran-Trio et al., 2021). This pattern might reveal hippocampus as a better brain region to diagnose CTD by ^1^H-MRS, and might reflect more dependency on CrT in order to maintain its Cr levels. In fact, such higher dependency on CrT was predicted in a rat brain expression analysis study since hippocampus was one of the brain regions with the highest percentage of CrT^+^ cells and with the lowest percentage of CrT^−^ cells co-expressing AGAT and GAMT (Braissant et al., 2010).

One of the essential CTD biomarkers in male patients is elevated urinary Cr/Crn ratio, which reflects the lack of Cr re-absorption from primary urine due to the lack of functional CrT in kidney proximal tubular cells. Significant increase in urinary Cr/Crn ratio was present in both *Slc6a8*^*Y*389*C*^ fHE and fHO rats. However, value intervals overlapped that from fWT ones, especially the fHE's ones, indicating that such biomarker might not be a preferred one for CTD diagnosis in female sex. This agrees with data from CTD female carriers, since only some of them showed elevated urinary Cr/Crn ratio (Nielsen et al., 2023). In contrast, urinary Cr concentration alone might still be an indicator heading to CTD diagnosis in female sex since ranges from *Slc6a8*^*Y*389*C*^ mutant female rats are separated from that of fWT rats.

Reduced body weight/slender build is a consistent feature of CTD pathology in male sex, in both preclinical and clinical studies (Ghirardini et al., 2021; van de Kamp et al., 2013; Miller et al., 2019). Supporting this data, fHO rats presented reduced body weight from young age. However, as occurs in KO heterozygous female mice (Hautman et al., 2014), body weight from fHE rats was not apparently affected by CrT deficiency, suggesting that the residual expression of the functional CrT (with a possible compensation increasing Cr synthesis) is sufficient to maintain normal body weight. This data is coherent with the general lack of attention to body weight/build in clinical reports of female CTD patients.

Muscle is one of the tissues with high energy demand and the main store of peripheral Cr in the body. Considering Cr roles (Hanna-El-Daher and Braissant, 2016; Sestili et al., 2016), both reduced myocyte size and low locomotor activity found in fHO rats are in agreement with the decrease in its muscular Cr levels. Interestingly, muscular GAA concentration was elevated in both fHE and fHO rats. These results might indicate an increase in endogenous Cr synthesis in order to compensate the uptake reduction caused by CTD, being sufficient to restore Cr levels in fHE rats.

Other hallmarks of CTD are cognitive deficiency and behavioral disturbances such as autistic behavior, attention deficits, hyperactivity, or aggressive behavior, among others (van de Kamp et al., 2013).

Such neurological phenotype generally appears stronger in male sex than in heterozygous female carriers, in both clinical and preclinical studies (Hautman et al., 2014; van de Kamp et al., 2013; Nielsen et al., 2023; Skelton et al., 2011). This data could point toward a correlation among the severity in the neurological phenotype, the lack of expression of functional CrT, and the depletion of brain Cr. Coherent with this hypothesis and data from a KO mice model (Hautman et al., 2014), *Slc6a8*^*Y*389*C*^ fHE rats presented lighter phenotype of cognitive deficiency than KI male rats, by not failing the working memory/attention task that KI male rats did (Duran-Trio et al., 2021). Additionally, fHE rats displayed a normal repetitive self-stimulating behavior (grooming) which was overdone in KI male rats (Duran-Trio et al., 2021).

Nevertheless, despite the total lack of functional CrT and the strong brain Cr deficiency in fHO rats, they exhibited normal performance in the same cognitive task and normal grooming behavior too. These results together might indicate that apart from zygosity/gene dosage, sex could have an important effect on CTD phenotype. Further studies are needed to delve in sex differences of CTD pathology, from proteomic and metabolomic profiles as already performed in other *in vitro* and *in vivo* models of CTD (Broca-Brisson et al., 2023; Disdier et al., 2024), to phenotypic traits and the exploration of the molecular mechanisms that underly physiology and behavior, in order to improve diagnosis and therapies for both sexes. In particular, markers of brain cell progenitors (SOX2, PAX6), neurogenesis (GSK3 β) and synapse development and function (postsynaptic marker PSD95, glutamate reuptake vGluT1transporter) should be investigated, as they have been shown altered in human brain organoid cultures issued of male CTD patients (Broca-Brisson et al., 2023). Markers of these specific steps of brain development and function are in correlation with our observations of alteration of cerebellum development in males of our *Slc6a8*^*Y*389*C*^ KI rat model (thinner molecular layer with lower density of Purkinje Cell dendritic spines, decrease of MAP2, NFM and pNFM neuronal markers, decrease of MBP oligodendrocyte marker and alteration of astrocytic structure; Duran-Trio et al., 2022). Brain glucose metabolism, which has been shown altered in other both *in vitro* and *in vivo* models of CTD (Disdier et al., 2024), should also be investigated in females and males of our *Slc6a8*^*Y*389*C*^ KI rat model.

On the other hand, fHE rats showed behavioral abnormalities such as altered behavioral response to stressors and a tendency to show increased aggressive behavior, which is in line with clinical data (Nielsen et al., 2023). Together these data highlight that anxiety-related behaviors (excessive or deficient) and hyperactivity might still be a CTD hallmark in female sex. Although strong behavioral disturbances could be observed spontaneously, common stressors could trigger altered behavioral responses too. This could be useful for CTD diagnosis although not every altered behavioral response could be labeled as pathogenic. It is worth noting that heterozygous female CTD carriers could benefit from Cr supplementation for symptomatic treatment (Bruun et al., 2018), improving their quality of life. It is particular true for CTD heterozygous female patients with mild to less severe neurological symptoms for whom one may speculate that blood-brain barrier still has a significant proportion of endothelial cells having active the × chromosome carrying the functional normal CRT. For CTD heterozygous female patients with more severe neurological symptoms for whom one may speculate that this proportion is much less, an interesting technique to develop for CTD deficiency could be the use of the × chromosome reactivation technology (XCR; Janiszewski et al., 2019). However, a lot of work would still be needed to target the right cells (blood-brain barrier, neurons).

Moreover, fHO rats also presented behavioral abnormalities, although there were zygotic differences in how rats behaved to certain stressors. Whether it is due to differences in locomotor activity alone (fading/developing some behavioral responses), and/or differences in the extent of their internal response to stressors or in the information processing is not known.

In summary, *Slc6a8*^*Y*389*C*^ heterozygous female rats present moderate/slight metabolic changes, normal body weight, apparently normal cognitive performance but behavioral abnormalities in response to stressors. This phenotype is coherent with the limited data from CTD female carriers, validating the *Slc6a8*^*Y*389*C*^ rat strain as a tool for the study of CTD pathology in female sex as well. These data also highlight that such phenotype could certainly be missed or underdiagnosed as CTD in female carriers, thus preventing them of the potential benefits of being treated by Cr supplementation.

X-chromosome inactivation studies are deciphering its mechanisms and its implications in the phenotypical diversity, slowly opening roads to understand and treat X-linked inherited diseases (Furlan and Galupa, 2022; Patrat et al., 2020). In CTD, there is still a long way to go to detangle the complexity of the combined effect of how mosaicism affects each body part (e.g., muscle or brain) and how such body parts interrelate among them resulting in a final phenotype (in terms of type, severity/penetrance, and the onset of each trait).

## Data Availability

The original contributions presented in the study are included in the article/Supplementary material, further inquiries can be directed to the corresponding author/s.

## Supplementary material

Supplementary Figure 1 **(A)** Boxplots of plasmatic and urinary metabolite concentrations. Pair comparisons are shown in the plots (Mann-Whitney test with Bonferroni correction). 15 fWT, 17 fHE and 14 fHO for plasmatic Cr and GAA levels; 11 fWT, 12 fHE and 10 fHO for plasmatic Crn levels; 15 fWT, 16 fHE and 15 fHO for urinary metabolite concentrations. **(B)** Boxplots of metabolite concentrations in cerebellum (Cb) and hippocampus (Hp) measured using ^1^H-MRS. Pair comparisons are shown in the plots (Tukey *post hoc* from ANOVA 1-way). 3 fWT, 8 fHE and 3 fHO. ^*^*P-value* < 0.05, ^**^*P-value* < 0.01, ^***^*P-value* < 0.001. Gln, Glutamine; Glu, Glutamate; NAA, N-Acetyl-Aspartate; NAAG, N-Acetyl-Aspartyl-Glutamate.

Supplementary Table 1 Summary of results from the behavioral tests used. Values from each genotype are shown as arithmetic mean with standard error of the mean.
