# Supplementary figures and images for: Heterozygous females from a rat model for creatine transporter deficiency reveal altered behavioral response to stressors, normal body weight and slight metabolic changes
Source: Front Neurosci. 2025 Apr 29;19:1520550. doi: 10.3389/fnins.2025.1520550 (PMC12070192; doi:10.3389/fnins.2025.1520550)

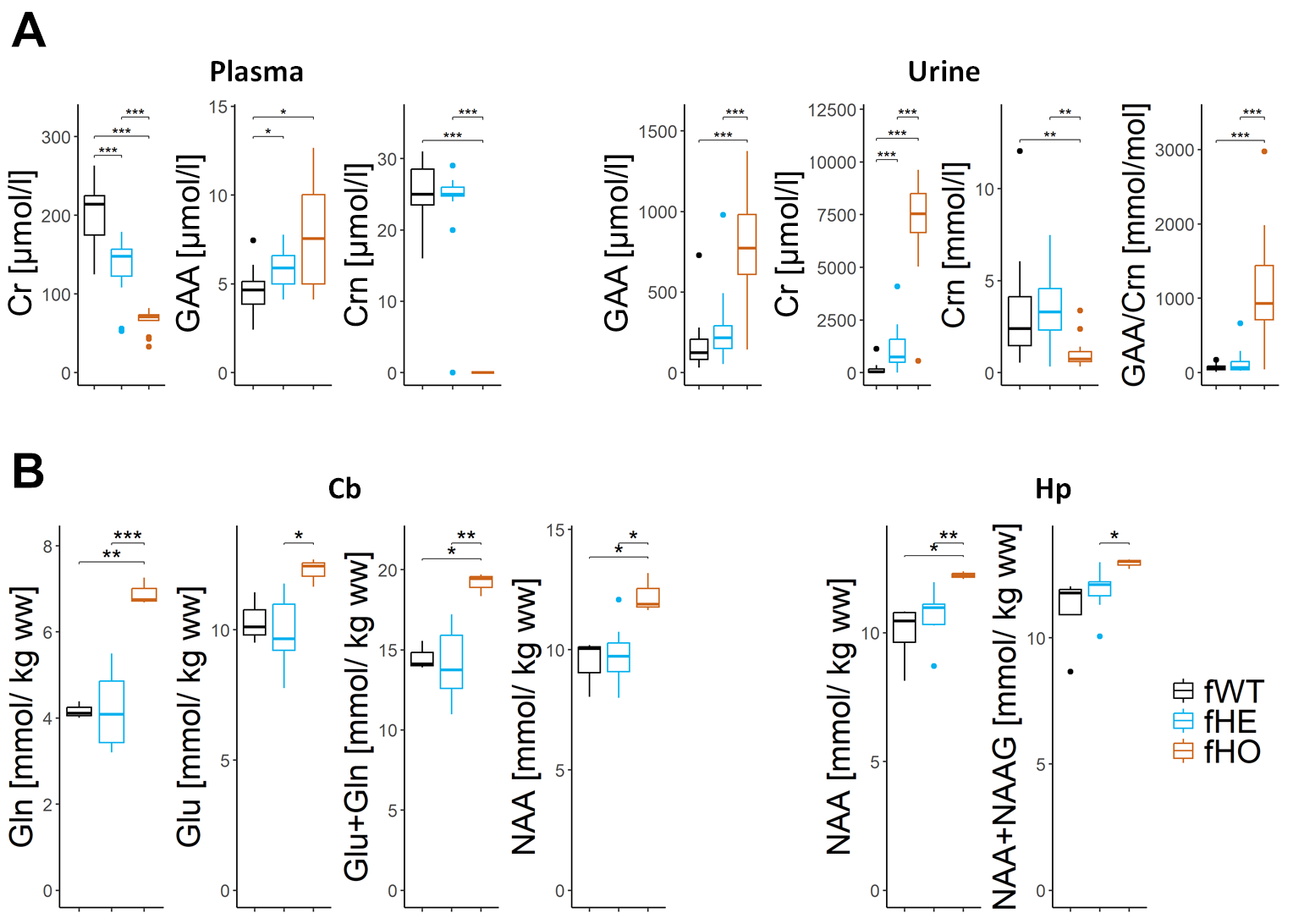

Supplement: Supplementary Figure 1 — (A) Boxplots of plasmatic and urinary metabolite concentrations. Pair comparisons are shown in the plots (Mann-Whitney test with Bonferroni correction). 15 fWT, 17 fHE and 14 fHO for plasmatic Cr and GAA levels; 11 fWT, 12 fHE and 10 fHO for plasmatic Crn levels; 15 fWT, 16 fHE and 15 fHO for urinary metabolite concentrations. (B) Boxplots of metabolite concentrations in cerebellum (Cb) and hippocampus (Hp) measured using 1H-MRS. Pair comparisons are shown in the plots (Tukey post hoc from ANOVA 1-way). 3 fWT, 8 fHE and 3 fHO. *P-value < 0.05, **P-value < 0.01, ***P-value < 0.001. Gln, Glutamine; Glu, Glutamate; NAA, N-Acetyl-Aspartate; NAAG, N-Acetyl-Aspartyl-Glutamate. [file Image_1.TIF]
